# Carbon‐13 Hyperpolarization of α‐Ketocarboxylates with Parahydrogen in Reversible Exchange

**DOI:** 10.1002/cmdc.202400378

**Published:** 2024-12-10

**Authors:** Stephen J. McBride, Keilian MacCulloch, Patrick TomHon, Austin Browning, Samantha Meisel, Mustapha Abdulmojeed, Boyd M. Goodson, Eduard Y. Chekmenev, Thomas Theis

**Affiliations:** ^1^ Department of Chemistry North Carolina State University 2620 Yarbrough Dr. Raleigh, NC 27695 USA; ^2^ Department of Physics North Carolina State University 2401 Stinson Dr. Raleigh, NC 27695 USA; ^3^ Vizma Life Sciences 400 South Elliot Rd., Suite D-178 Chapel Hill, NC 27514 USA; ^4^ School of Chemical & Biomolecular Sciences Materials Technology Center Southern Illinois University 1245 Lincoln Dr. Carbondale, IL 62901 USA; ^5^ Integrative Biosciences Department of Chemistry Karmanos Cancer Institute Wayne State University 5101 Cass Ave. Detroit, MI 48202 USA

**Keywords:** Hyperpolarization, NMR, SABRE, Alpha-ketocarboxylate, Parahydrogen

## Abstract

Signal Amplification by Reversible Exchange (SABRE) is a relatively simple and fast hyperpolarization technique that has been used to hyperpolarize the α‐ketocarboxylate pyruvate, a central metabolite and the leading hyperpolarized MRI contrast agent. In this work, we show that SABRE can readily be extended to hyperpolarize ^13^C nuclei at natural abundance on many other α‐ketocarboxylates. Hyperpolarization is observed and optimized on pyruvate (P_13C_=17 %) and 2‐oxobutyrate (P_13C_=25 %) with alkyl chains in the R‐group, oxaloacetate (P_13C_=11 %) and alpha‐ketoglutarate (P_13C_=13 %) with carboxylate moieties in the R group, and phenylpyruvate (P_13C_=2 %) and phenylglyoxylate (P_13C_=2 %) with phenyl rings in the R‐group. New catalytically active SABRE binding motifs of the substrates to the hyperpolarization transfer catalyst – particularly for oxaloacetate – are observed. We experimentally explore the connection between temperature and exchange rates for all of these SABRE systems and develop a theoretical kinetic model, which is used to fit the hyperpolarization build‐up and decay during SABRE activity.

## Graphical abstract

Hyperpolarization enhances NMR and MRI signals of small molecules at low concentrations to enable new applications in chemical analysis and molecular imaging. This work demonstrates that the broad class of alpha‐ketocarboxylates can be hyperpolarized by parahydrogen in catalyzed reversible exchange reactions. Experimental insights regarding the exchange dynamics and refined theoretical models are provided to optimize the achievable hyperpolarization.

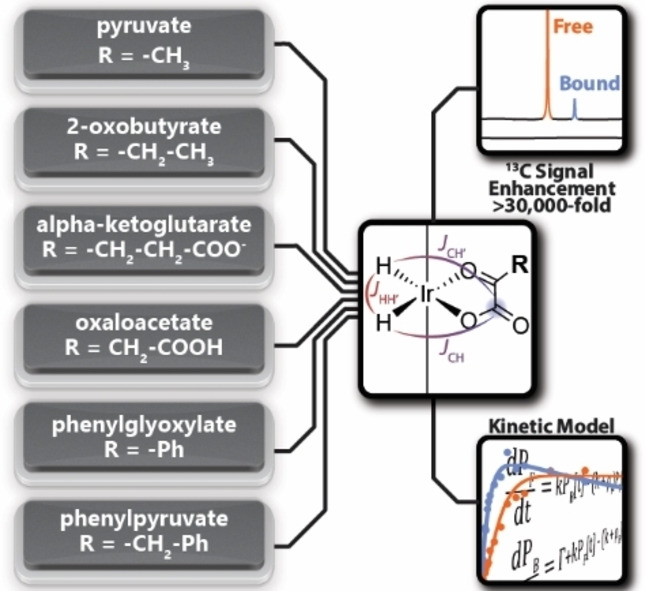

## 1 Introduction

Magnetic Resonance (MR) techniques grant exceptional insights into molecular structure and dynamics.[^1^, ^2^, ^3^, ^4^, ^5^, ^6^] However, due to the relatively small energy difference between spin states and associated low thermal polarization on nuclear spins,^[7]^ (especially for nuclear spins with low gyromagnetic ratio such as ^13^C) the full potential of MR techniques such as Nuclear Magnetic Resonance (NMR) and Magnetic Resonance Imaging (MRI) remains unexploited. For example, most clinical MRI applications are limited to anatomical imaging of water and fat *in vivo*, and metabolites at lower concentrations cannot be easily imaged at magnetic fields of standard MRI scanners (~3 T).[^8^, ^9^, ^10^] However, emerging hyperpolarization techniques can overcome the sensitivity limits of conventional NMR and MRI techniques by enhancing nuclear spin polarization towards unity.[^11^, ^12^] Since NMR signal is directly proportional to the nuclear spin polarization (*P*), the use of hyperpolarization can increase NMR signal by several orders of magnitude. Leading hyperpolarization modalities include Dynamic Nuclear Polarization (DNP),[^13^, ^14^, ^15^, ^16^] Spin‐Exchange Optical Pumping (SEOP),^[17]^ and Parahydrogen‐Induced Polarization (PHIP).[^18^, ^19^, ^20^] DNP[^21^, ^22^, ^23^, ^24^, ^25^, ^26^] and SEOP[^27^, ^28^, ^29^, ^30^] have led the charge towards clinical translation of hyperpolarized MRI (HP MRI). Recently, a SEOP‐based method for hyperpolarizing ^129^Xe gained FDA approval for lung imaging with MRI.^[31]^ DNP techniques have excelled in hyperpolarization on a wide range of substrates and have shown promising results in Phase 1 clinical trials.[^21^, ^22^, ^23^, ^24^, ^25^, ^26^, ^32^, ^33^, ^34^, ^35^, ^36^] The large majority of DNP hyperpolarized MRI experiments are conducted with [1‐^13^C]pyruvate because it is a central energy metabolite and has rapid cellular uptake and reactivity.^[37]^ Hyperpolarized pyruvate has found use for disease characterization in a wide variety of oncology[^33^, ^37^] and cardiac[^38^, ^39^] applications. Nonetheless, concerns associated with high costs (~$2 M device cost), slow agent throughput due to long polarization buildup times, and large instrument footprint have limited the adoption of DNP‐enabled imaging studies.

**Figure 1:**
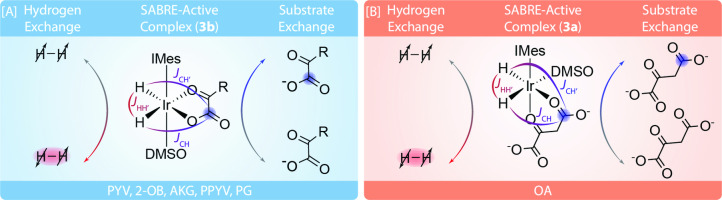
SABRE reaction scheme demonstrating the α‐ketocarboxylate substrate and *p*‐H_2_ reversibly binding to the Ir‐IMes catalyst in the presence of DMSO to form the primary SABRE catalytically active complex for each metabolite ((A) **3 b** for pyruvate (PYV), 2‐oxobutyrate (2‐OB), alpha‐ketoglutarate (AKG), phenylpyruvate (PPYV), and phenylglyoxylate (PG), and (B) **3 a** for oxaloacetate (OA)). IMes=1,3‐bis(2,4,6‐trimethylphenyl)imidazol‐2‐ylidene). R=−CH_3_ for PYV, −CH_2_−CH_3_ for 2‐OB, −CH_2_−CH_2_−COO^−^ for AKG, −Ph for PG, and −CH_2_−Ph for PPYV. The SABRE‐active complexes are referred to as **3 a** and **3 b** by the convention established in Tickner et al. (2020).^[59]^

In contrast, PHIP and its non‐hydrogenative modality Signal Amplification By Reversible Exchange (SABRE)^[40]^ have emerged as cheap, rapid, and simple hyperpolarization alternatives that are suitable for *in vivo* MRI.[^41^, ^42^, ^43^] The SABRE method relies on the reversible exchange of parahydrogen (*p*‐H_2_) and a suitable substrate on an Ir metal catalyst. When both *p*‐H_2_ and substrate are bound to the catalyst, a temporary *J*‐coupling network is formed between the magnetically inequivalent parahydrogen‐derived hydrides and a target nucleus on the substrate. Through magnetic field control, spin order can then be transferred from the *p*‐H_2_ singlet state directly to a clinically relevant substrate without chemical modification, as shown in Figure 1. For proton SABRE, the optimal magnetic field is on the order of 6 mT. Both *p*‐H_2_ and hyperpolarized substrate experience reversible exchange, resulting in continuous buildup of hyperpolarized substrate in solution at this specific magnetic field, which is responsible for energy‐level matching, allowing for flow of polarization from hydrides to substrate.

The scope of SABRE chemistry has expanded considerably since its first demonstration in 2009, optimized for the hyperpolarization of nuclear spins in pyridine and related substrates – including hyperpolarization of ^1^H and heteronuclei (^13^C, ^15^N, ^31^P, etc).^[40]^ Subsequent works employed SABRE to hyperpolarize additional pyridine derivates,[^40^, ^44^] nitriles,[^45^, ^46^, ^47^] and other nitrogen‐containing heterocycles.[^48^, ^49^, ^50^, ^51^, ^52^, ^53^] In 2015, a related technique called SABRE in SHield Enables Alignment Transfer to Heteronuclei (SABRE‐SHEATH) was introduced to directly transfer spin order to heteronuclei but was still limited to compounds containing nitrogen atoms capable of binding to the SABRE catalyst.[^54^, ^55^] In SABRE, hyperpolarization transfer of hydride polarization to target nuclei requires matching of the frequency difference between polarization source and target to the hydride‐hydride *J*‐coupling. For proton SABRE, this matching occurs at ~6 mT. Whereas for heteronuclei e. g. ^15^N, ^13^C (SABRE‐SHEATH), the matching occurs at magnetic fields ~0.2–1.0 μT, which are easily obtained inside of magnetic shields. The efficient hyperpolarization of heteronuclei (^15^N and ^13^C) is valuable because these nuclei are associated with much slower relaxation times than protons, are free from background signals, and can exhibit large frequency shifts induced by chemical reactions or changes in the local biochemical environment.

In 2019, novel developments unlocked a greater substrate scope, enabling hyperpolarization of ^13^C sites on pyruvate and other α‐ketocarboxylates.^[56]^ The hyperpolarization of these substrates, including pyruvate, required the introduction of co‐ligands, such as dimethyl sulfoxide (DMSO) to modulate kinetics.[^56^, ^57^, ^58^, ^59^] The ability to hyperpolarize [1‐^13^C]pyruvate enables SABRE to follow in the footsteps of existing pre‐clinical and clinical work, while promising more scalable technology.[^40^, ^41^, ^42^, ^43^, ^56^, ^57^, ^58^, ^59^] While pyruvate has paved the way for HP MRI, boasting over 30 ongoing clinical trials, other α‐ketocarboxylates are attractive compounds for HP MRI because many of them also have important roles in mammalian metabolism.^[60]^

In this work, we demonstrate that SABRE chemistry is widely applicable to the broad class of α‐ketocarboxylates. Specifically, we investigated SABRE‐SHEATH hyperpolarization of pyruvate (PYV), 2‐oxobutyrate (2‐OB), alpha‐ketoglutarate (AKG), phenylpyruvate (PPYV), phenylglyoxylate (PG), and oxaloacetate (OA). 2‐OB and its metabolic derivatives have been implicated as key metabolites in identifying colorectal cancers.^[61]^ Hyperpolarized AKG prepared by d‐DNP has been used to inform upon altered metabolism resulting from genetic mutations related to glioblastomas^[62]^ and has recently been hyperpolarized with SABRE.^[63]^ PPYV and its downstream metabolites are present in the blood and urine of infants with phenylketonuria.^[64]^ PG has been identified as the terminal metabolite of the volatile organic compound styrene, which is sometimes inhaled or ingested and can be found in packaging made of polystyrene.^[65]^ Additionally, PG shares a metabolic pathway with the antipsychotic haloperidol.^[65]^ Finally, OA has been used to accelerate and improve bone growth and regeneration in mice.^[66]^

This paper not only expands the SABRE substrate scope to several new biologically relevant molecules, but also uses the chemical diversity of these systems to shed light on SABRE dynamics and mechanism of action. Specifically, we performed polarization dynamics studies, including polarization buildup and relaxation, for each α‐ketocarboxylate. We then derived an analytical model that connects substrate exchange, polarization buildup, and *T*_1_ relaxation of these α‐ketocarboxylates. The present work shows that virtually all α‐ketocarboxylates can be hyperpolarized by SABRE. The hyperpolarization of this class of molecules is found to be very robust and generally works at the first attempt when following the presented procedures. The derived analytical model used to fit the presented data sheds light on the substrate kinetics and spin exchange dynamics and ultimately will help to further broaden the SABRE‐substrate scope in the future.

## Methods

### Chemicals

Methanol‐*d_4_* was purchased from Cambridge Isotope Laboratories. Sodium pyruvate, disodium alpha‐ketoglutarate, sodium phenylpyruvate, sodium 2‐oxobutyrate, oxaloacetic acid, phenylglyoxylic acid, sodium hydroxide, and DMSO were purchased from Millipore Sigma. Methanol‐*d*_4_ and DMSO were degassed using a freeze‐pump‐thaw method and stored under argon at room temperature. The SABRE pre‐catalyst, [Ir(IMes)(COD)Cl] (COD=1,5‐cyclooctadiene, IMes=1,3‐bis(2,4,6‐ trimethylphenyl)imidazol‐2‐ylidene) was synthesized in house according to prior published work^[67]^ with commercially available reagents.

### Sample Preparation

Solutions were prepared by dissolving 6 mM SABRE pre‐catalyst, 30 mM α‐ketocarboxylate substrate, and 24 mM DMSO in 600 μL of methanol‐*d*_4_ under argon in a 3 mL scintillation vial. For solutions involving α‐ketocarboxylates in their acidic form (OA and PG), a 1 : 1 ratio of NaOH in methanol‐*d*_4_ was added to deprotonate the carboxylic acid group. This step resulted in the addition of H_2_O in a 1 : 1 H_2_O‐to‐substrate ratio, which has been previously shown to have a minor impact on PYV polarization at small water concentrations.^[58]^ The scintillation vial was then sonicated until all solids were fully dissolved. 600 μL of each sample was then transferred to a high‐pressure, 7” length, 5 mm OD NMR tube (SP Wilmad‐LabGlass 524‐PV‐7) under argon. Following sample preparation, the samples were connected to our in‐house pneumatic shuttling system,^[68]^ where they were pressurized to 100 psi with parahydrogen.

### Temperature Dependence

To obtain temperature‐dependent measurements, the samples were lowered into the bore of a Bruker Ascend 9.4 T NMR with Neo Advance III console. 93 % *p*‐H_2_ was bubbled into the samples for 10 minutes at a flow rate of 75 sccm and 25 °C to activate the catalyst. After activation, the sample temperature was reduced to −10 °C. The sample was then shuttled out of the magnet and into a polarization transfer field (PTF) set to 0.3 μT where it was no longer actively cooled and began to equilibrate with ambient temperatures. While in the PTF, *p*‐H_2_ was bubbled into the sample for 90 s at 100 psi and 75 sccm. The sample was shuttled back into the bore of the magnet, where a 90° pulse was applied followed bysignal detection. After detection, the temperature was adjusted, and the sample temperature was equilibrated before the next experiment.

### *T*_1_ Lifetimes

To obtain *T*_1_ lifetimes, the samples were cooled to 0 °C (−10 °C for PGA) in the bore of the magnet. The sample was then shuttled into the PTF set to 0.3 μT where it was no longer actively cooled and began to equilibrate with ambient temperatures. While in the PTF, *p*‐H_2_ was bubbled into the sample for 90 s (15 s for PGA) at 100 psi and 75 sccm. After a variable delay with the sample remaining at 0.3 μT, the sample was shuttled back into the bore of the magnet for signal detection at high field.

### Polarization Buildup

To obtain polarization buildup measurements, the samples were cooled to 0 °C (−10 °C for PGA) in the bore of the magnet. The sample was then shuttled out of the magnet into the PTF set to 0.3 μT and *p*‐H_2_ (100 psi and 75 sccm) was bubbled into the sample for a variable amount of time. The *p*‐H_2_ flow was stopped, and the sample was shuttled into the bore of the magnet for signal detection at high field.

### Polarization Calculations

Polarization values were determined using pure [2‐^13^C]‐ethyl acetate thermalized at 9.4 T as a reference following a previously reported method as detailed in the SI (S2–3).^[54]^

## 2 Results

### 2.1 The Range of Enabled α‐Ketocarboxylates and Maximum Hyperpolarization Values

**Figure 2:**
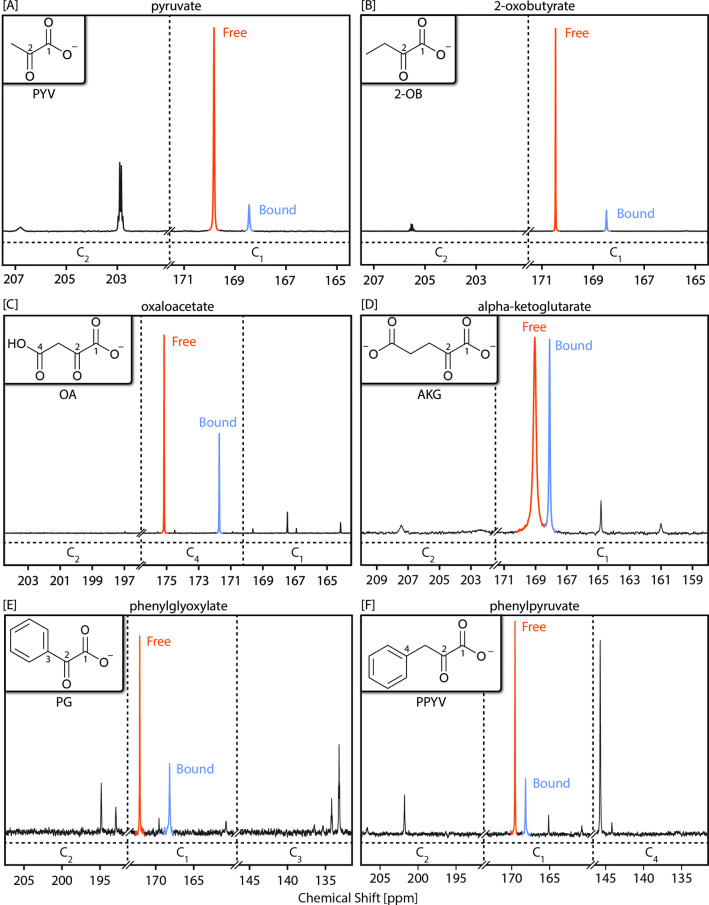
Representative hyperpolarized ^13^C NMR spectra for each natural abundance α‐ketocarboxylate examined in this work. Each spectrum is the result of a single scan using a Bruker Ascend 9.4 T NMR spectrometer. The spectra are scaled for easier comparison. Peaks are labelled by section according to their position (lower) and whether they are free in solution (orange) or bound to the polarization transfer catalyst (blue). The additional peaks are currently unassigned and further work is needed to identify them. The color‐labelled peaks are those used for the remainder of analysis in this work. Peaks were labelled based on prior published works for pyruvate (PYV),^[56]^ oxaloacetate (OA),^[69]^ alpha‐ketoglutarate (AKG),^[63]^ observed chemical exchange, temperature dependence, and polarization relaxation/buildup dynamics.

SABRE‐SHEATH hyperpolarized ^13^C spectra for PYV, 2‐OB, OA, AKG, PG and PPYV are presented in Figure 2A–F. The maximum obtained hyperpolarization levels are given in Table 1. The α‐ketocarboxylates in this list are ordered by structural similarities in the alpha‐keto R‐groups.

**Table 1:** A summary of maximum ^13^C polarization observed for each nucleus across all experiments. Blanks indicate no observed signal enhancements.

Substrate		C_1_ Polarization (%)		C_2_ Polarization (%)		C_3_ Polarization (%)		C_4_ Polarization (%)	
		Free	Bound	Free	Bound	Free	Bound	Free	Bound
PYV	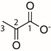	16.9	15.2	7.0	4.2	0.3	–	–	–
2‐OB	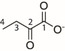	19.9	24.9	2.3	2.1	<0.1	–	0.1	–
OA	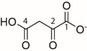	–	–	<0.1	–	–	–	5.2	10.9
AKG	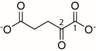	6.6	13.3	0.3	–	–	–	–	–
PG	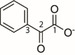	0.7	2.0	0.4	0.6	0.7	0.7	–	–
PPYV	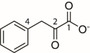	1.3	2.0	0.4	–	–	–	1.8	0.4

Polarization is primarily observed on C_1_ for most α‐ketocarboxylates. Under the experimental conditions used in this work, peak C_1_ polarization values for PYV in its bound and free forms are 15.2 % and 16.9 %, respectively. For 2‐OB, we observe 24.9 % bound C_1_ polarization and 19.9 % free C_1_ polarization. For OA, surprisingly C_4_ exhibits the highest polarization, corresponding to values of 10.9 % and 5.2 % for bound and free forms, respectively. AKG bound C_1_ polarization was 13.3 %, whereas free C_1_ polarization was 6.6 %. The phenyl‐substituted substrates exhibit maximum polarization on bound C_1_ (2 %) but show comparable polarization across all carbon nuclei in their chain (C_1_−C_4_). For PG, we note a bound C_1_ polarization of 2.0 % and a free C_1_ polarization of 0.7 %. PPYV shows a bound C_1_ polarization of 2.0 % and a free C_1_ polarization of 1.3 %. The remainder of this work focuses on C_4_ polarization for OA and C_1_ polarization for all other substrates.

The catalytically active species for most α‐ketocarboxylates is the equatorial bidentate isomer (**3 b**),^[59]^ where the substrate is in plane with the parahydrogen‐derived hydrides (Figure 1A).^[59]^ However, OA likely uses a primarily equatorial‐axial bidentate isomer (**3 a**)^[59]^ in which the oxygen on the C_4_ and C_2_ bind to the iridium metal center to form a 6‐membered ring (Figure 1B). This claim is supported by the fact that the chemical shift separation between C_1_ resonances of **free** PYV (169.7 ppm), **3 b‐bound** PYV (168.5 ppm), and **3 a‐bound** PYV (165.2 ppm) matches the chemical shift separation pattern of C_4_ for **free** OAA (175.2 ppm), **3 b‐bound** OA (174.5 ppm), and **3 a‐bound** OA (171.7 ppm). However, the primary polarization of the bound OA is observed on the **3 a** species. (See Figure 2A and C). As illustrated in the SI (S4–5), OA in its deprotonated form and in the presence of metals is known to decarboxylate to form PYV and CO_2_,^[69]^ preventing the accurate characterization of *T*_1_ relaxation and polarization. To avoid overreporting OA polarization, all polarization values for OA are based on the original substrate concentration (see SI (S2–3)).

### 2.2 Temperature Dependence

**Figure 3:**
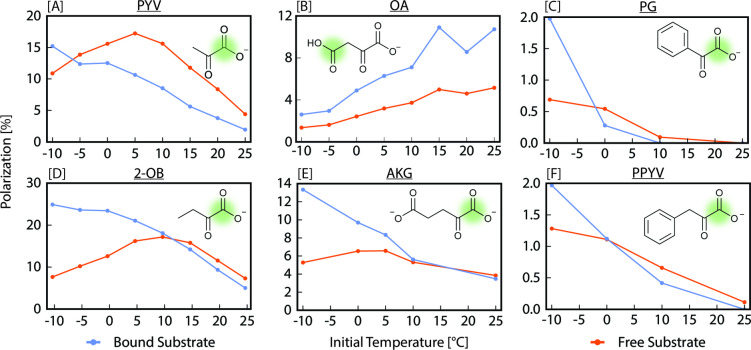
^13^C polarization of specified nucleus (highlighted in green) for each α‐ketocarboxylate in its free (orange) and bound (blue) forms as a function of initial sample temperatures ranging between −10 °C and 25 °C with 90 s of parahydrogen bubbling.

To characterize the SABRE dynamics and understand how an assortment of α‐ketocarboxylates compares to the well‐probed PYV case, we examined C_1_ polarization of the free and bound substrate (C_4_ for OA) across a range of initial temperatures in a series of temperature‐cycling experiments. Initial temperature refers to the sample temperature at the beginning of the hyperpolarization process. During p‐H_2_ bubbling, hyperpolarization builds up and the sample equilibrates with ambient conditions. The corresponding temperature‐dependent profiles are reported in Figure 3. As reported in previous temperature‐cycling experiments,^[57]^ PYV (Figure 3A) exhibited maximum polarization on the catalyst‐bound substrate at the lowest temperature examined (−10 °C) and decreased steadily with increasing temperature. All other substrates investigated, aside from OA, exhibited the same trend. This result indicates that at low temperature, substrate exchange is slow while the hydride exchange remains sufficiently fast for spin order transfer to efficiently occur. As seen in Figure 3A, free PYV polarization begins well below its maximum at low temperature and peaks at an initial temperature of 5 °C. Polarization then begins to rapidly decrease as initial temperature increases. 2‐OB (Figure 3D) and AKG (Figure 3E) follow this trend with maximum polarization on the free substrate at 10 and 5 °C, respectively, indicating the initial temperature at which the interplay between substrate and hydride exchange is optimal for hyperpolarization buildup on the free substrate under the given experimental conditions. Conversely, PG (Figure 3C) and PPYV (Figure 3F) exhibit maximum polarization on the free substrate at the lowest initial temperature examined (−10 °C), which rapidly decreases with initial temperature. While at first the temperature profiles of PG and PPYV appear dissimilar to the PYV case, it is likely that exchange rates necessary for most efficient spin order transfer occur for PG and PPYV at lower temperatures than those available on our instrumentation for this work. Notice that Figure 3C and F indicate that free (and bound) polarization may be increased further at even lower initial temperatures.

Unlike PYV or any other substrate examined in this work, the free and bound polarization values for OA (Figure 3B) retained the same ratio across the examined initial temperatures. Also unlike all other substrates, OA showed an increase in polarization of both free and bound substrate with increasing temperature, peaking at the highest temperature examined. This result indicates an increase in parahydrogen‐derived hydride exchange rates at elevated temperatures with a limited impact on substrate exchange rates. As OA readily decarboxylates under SABRE conditions, further studies should be performed to confirm this hypothesis.^[69]^ An examination of the temperature‐dependent data in comparison to PYV provides an efficient method for determining relative exchange rates at various temperatures –as we detail in the following sections using a two‐state model of differential equations.

### 2.3 *T*_1_ Relaxation

**Figure 4:**
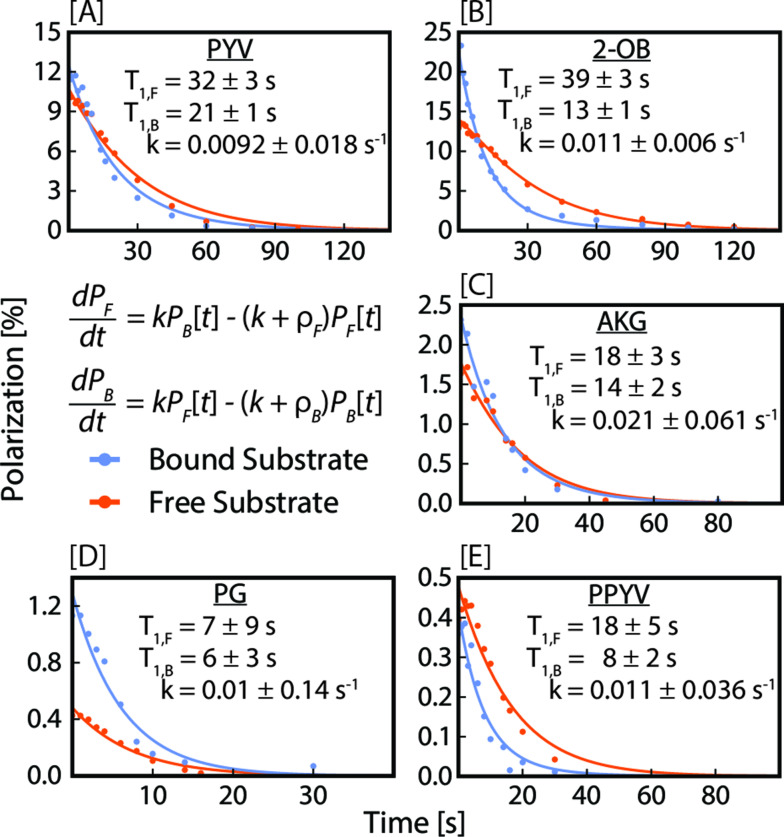
Low‐field (0.3 μT) ^13^C *T*_1_ relaxation rates and fits for the bound (blue) and free (orange) forms of each substrate (n=1) after 90 s of *p*‐H_2_ bubbling at an initial temperature of 0 °C (15 s and −10 °C for PG). OA has been omitted from this figure as it readily decarboxylates under SABRE conditions, preventing the accurate characterization of *T*_1_ relaxation.

Following our previous work,^[57]^ we fit the low‐field *T*_1_ relaxation data to a two‐state (free‐bound) system of differential equations,

(1) $$\frac{dP_{F}}{dt}=kP_{B}\left[t\right]-\left(k+\rho_{F}\right)P_{F}\left[t\right]\;$$

(2) $$\frac{dP_{B}}{dt}=kP_{F}\left[t\right]-\left(k+\rho_{B}\right)P_{B}\left[t\right]\;$$

where *P*_F_ and *P*_B_ are the C_1_ polarization of the free and bound species, *ρ*_F_ and *ρ*_B_ are the relaxation rates of the free and bound species, and *k* is the exchange rate between the α‐ketocarboxylate and the Ir‐IMes catalyst. The raw data plotted against the fits is displayed in Figure 4. To build a more accurate relaxation model, we initially fit the free and bound relaxation data to a mono‐exponential decay function,

(3) $$P_{x}\left[t\right]=P_{0}e^{\frac{-\left(t-t_{0}\right)}{T_{1}}}\;$$

These fitted mono‐exponential decay functions were used to extract the initial free and bound polarization values, which were subsequently used as boundary conditions for fitting the analytical solution of the set of differential equations to the data. This procedure allowed us to extract the relaxation rates *ρ*_F_ and *ρ*_B_, of the free and bound substrate as well as the exchange rate constant *k*. All the fits and the extracted values are displayed in Figure 4. We expect a bound substrate to have a shorter *T*_1_ than a free substrate due to both an increase in spin‐coupling partners at microtesla fields and longer correlation times when bound to the catalyst. When the observed *T*_1_ values are close, then the extracted exchange rate is associated with a relatively large error. This issue arises because in the limit of identical relaxation rates of free and bound substrate, any exchange rate would lead to a good fit to the data. Nonetheless, it is noteworthy that the extracted exchange rates for all substrates are close to each other within the associated errors. Note that the exchange rates are very small compared to those optimized for the SABRE hyperpolarization of ^15^N in substrates such as pyridine or acetonitrile.[^46^, ^53^] For the α‐ketocarboxylates studied here, we observed exchange rates ranging from 0.0092 s^−1^ for PYV (Figure 4A) to 0.021 s^−1^ for AKG (Figure 4C), corresponding to average complex lifetimes of 109 s and 48 s, respectively. These average lifetimes appear very long but can be rationalized by the small hydride‐^13^C *J*‐couplings present in these systems. These hydride‐^13^C *J*‐couplings are on the order of tens to hundreds of mHz, which require slow exchange rates for efficient hyperpolarization transfer because of the associated slow spin evolution – hence the need for lower temperatures and longer *p*‐H_2_ bubbling durations to achieve higher substrate polarization values.

Similar to previous studies,^[57]^ PYV (Figure 4A) exhibits *T*_1_ relaxation time constants of 32±3 s and 21±1 s for free and bound substrate, respectively, at microtesla fields, with a corresponding exchange rate of 0.0092±0.018 s^−1^. Similarly, 2‐OB (Figure 4B) exhibits free and bound *T*_1_ values of 39±3 s and 13±1 s, respectively, with an exchange rate of 0.011±0.006 s^−1^. The addition of the CH_2_ group decreases spin‐coupling partners for 2‐OB, resulting in a slight elongation of *T*_1_ relaxation for 2‐OB in comparison to PYV. In contrast to PYV, PG and PPYV (Figure 4D and E) exhibit faster free and bound *T*_1_ relaxation times. However, the exchange rates for PGA and PPYV closely match those of PYV and 2‐OB.

Although the *T*_1_ relaxation times of these substrates are relatively short at microtesla fields, it has been shown that lifetimes can be extended by operating at intermediate fields (~1 T), which reduces the respective impacts on relaxation of chemical shift anisotropy (more efficient at higher fields) and dipolar coupling (more efficient at microtesla fields).[^7^, ^48^, ^51^, ^52^, ^70^, ^71^, ^72^] Additionally, recent works have shown that a combination of deuteration schemes and multi‐axial, adiabatic pulse sequences can substantially increase *T*_1_ relaxation times for pyruvate and could be applied to these similar substrates.[^42^, ^72^]

### 2.4 Polarization Buildup

To model polarization buildup, a separate set of differential equations describing the same two‐state (free‐bound) system is employed – with the addition of a polarization pumping term, Γ, to the free polarization equation to describe the transfer of spin‐order from *p*‐H_2_ to the substrate:

(4) $$\frac{dP_{F}}{dt}=kP_{B}\left[t\right]-\left(k+\rho_{F}\right)P_{F}\left[t\right]\;$$

(5) $$\frac{dP_{B}}{dt}=\Gamma +kP_{F}\left[t\right]-\left(k+\rho_{B}\right)P_{B}\left[t\right]\;$$

Γ is derived from the level anti‐crossing of a 3‐spin system, here between the two parahydrogen‐derived hydrides and the primarily C_1_ target spin of the α‐ketocarboxylate. The spin‐order transfer from the parahydrogen‐derived hydrides to the C_1_ of the α‐ketocarboxylate is described by the blocks of the Hamiltonian that connect the parahydrogen‐derived hydride $\left|S_{0}\right.\rangle$ state and carbon |α⟩ state.^[70]^ Based on previous work,^[70]^ we rederived the buildup of the |α⟩ state on the carbon in a 3‐spin model as detailed in the SI (S9–10) to obtain:

(6) $$\Gamma =\frac{\pi^{2}\tau_{life}\Delta J_{CH}^{2}}{1+\pi^{2}\tau_{life}^{2}\left(2\Delta J_{CH}^{2}+4{\left(-J_{HH}+\frac{\Sigma J_{CH}}{4}+\Delta \nu_{CH}\right)}^{2}\right)}$$

Where $\tau_{life}$ is the lifetime of the parahydrogen‐derived hydrides, $J_{HH}$ is the *J*‐coupling between the parahydrogen‐derived hydrides, 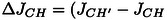 ), 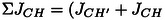 ), and $\Delta \nu_{CH}=\left(\nu_{H}-\nu_{C}\right)$ .

As the lifetime of the parahydrogen‐derived hydrides (τ_life_) is temperature‐dependent and the solution temperature changes over the course of the experiment, an Arrhenius equation was used, where *p*, *a*, and *g* are fitting parameters:

(7) $$\tau_{life}\left[t\right]=\frac{1}{k_{hydride}}=\frac{1}{p+a\cdot e^{\frac{-g}{T\left[t\right]}}}$$

**Figure 5:**
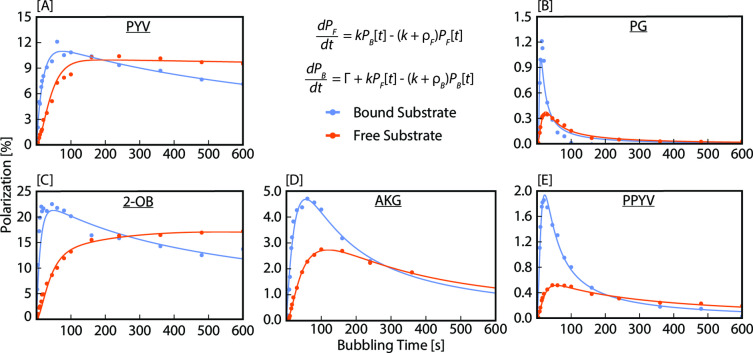
Polarization for each α‐ketocarboxylate substrate in the bound (blue) and free (orange) forms as a function of parahydrogen bubbling time. Data are fit to the system of differential equations described in Eqs. 4–7 and the resulting curves are presented. As bubbling time increases, sample temperature continues to equilibrate with ambient conditions. The in‐depth solution to the differential equations and resulting fitting parameters are presented in the SI (S11). OA has been omitted from this figure as it readily decarboxylates under SABRE conditions, preventing the accurate characterization of polarization buildup.

This set of differential equations with pumping term (Eqs. 4–5) was then given the boundary conditions that initial free and bound polarization are both zero, and then was analytically solved. The resulting functions are then fit to the experimental free and bound polarization buildup data, using the free and bound relaxation rates ($\rho_{F}$ ,$\;\rho_{B}$ **)** and exchange rate (*k*) determined from the *T*_1_ relaxation data. The raw buildup data plotted against the fitted functions is found in Figure 5. A summary of the fitting parameters (*p*, *a*, *g*) can be found in the SI (S11).

The fits show a rapid buildup of polarization on the bound substrate as the temperature is low and substrate exchange rate is slow. As the sample increases in temperature (along with hydride exchange rate), bound polarization quickly peaks and begins to decrease while free polarization continues to increase slowly. Our additional data on temperature dependent linewidths of hydride and ^13^C‐substrate, presented in the SI (S12–13), shows that the hydride exchange rates are faster than the substrate exchange rates. The hydride exchange rate also has a larger temperature dependance than the substrate exchange rate, which is why we initiate the hyperpolarization at low temperature to hyperpolarize the bound substrate first and then drive release of the substrate into its free form at elevated temperatures.

Like PYV (Figure 5A), 2‐OB (Figure 5C), achieves maximum polarization on the free substrate at approximately 150 seconds, suggesting similar exchange rates. In contrast, AKG (Figure 5D), PG (Figure 5B), and PPYV (Figure 5E) achieve maximum polarization on the free substrate much more quickly than PYV, at 90 s for AKG, 15 s, for PG and 50 s,for PPYV. To understand this behavior and the differences between PYV and 2‐OB vs. AKG, PG, and PPYV more closely, we also examined the linewidths of the hydride resonances, measured at Full Width Half Maximum (FWHM). This hydride linewidth can be used to assess the hydride exchange rate.^[70]^ For AKG, PG, and PPYV the hydride linewidths and the associated exchange rates are larger and increase more rapidly with temperature than for PYV and 2‐OB (see SI (Figure S3)). As faster exchange leads to broader linewidths,^[70]^ the observed temperature dependencies suggest that hydride exchange rates for AKG, PG, and PPYV at elevated temperatures are too rapid to allow for sufficient polarization transfer. We suspect that the bidentate binding modes in combination with the larger steric hindrance found in AKG, PG, and PPYV destabilize the hyperpolarization transfer catalyst leading to the elevated hydride exchange.. Note that fast hydride exchange rates, as compared to the rate of *J*‐coupling‐mediated polarization transfer (where $J_{CH}\ll k_{hydride}$ ), leads to inefficient polarization. Consequently, bound polarization decreases quickly with increasing temperature for these substrates as compared to PYV and 2‐OB, and the overall polarization for these three compounds, AKG, PG and PPYV, remains lower. Therefore, optimal conditions for α‐ketocarboxylate polarization likely depend on operating in a regime in which hydride exchange rates remain sufficiently slow to allow for polarization transfer while ensuring a sufficiently fast substrate exchange rate to build free polarization. In summary, the combination of the experimental data with the fitted model and the information on exchange rates extracted from the linewidth analysis, give detailed insight into the behavior of these α‐ketocarboxylate systems and guide future optimization of hyperpolarization levels.

## 3 Conclusions

In this paper, we have demonstrated the efficient SABRE hyperpolarization of six α‐ketocarboxylates using SABRE‐SHEATH, marking the first SABRE‐SHEATH hyperpolarization of four of these substrates: 2‐OB, OA, PPYV, and PG. Using this diverse set of α‐ketocarboxylate biomolecules, we conclude that this class of molecules have similar binding modes to the SABRE catalyst, enabling high levels of ^13^C polarization for PYV (*P*_13C_=17 %), 2‐OB (*P*_13C_=25 %), and AKG (*P*_13C_=13 %) on C_1_. When the substrate is equipped with another carboxylate moiety, such as is the case for OA, catalyst binding can be different, leading to efficient hyperpolarization of C_4_ (*P*_13C_=11 %) instead. This finding is remarkable as it may lead to the development of new binding schemes for SABRE hyperpolarization of other classes of substrates. The substrate functionalization with bulky phenyl groups results in less favorable catalyst binding dynamics, in turn reducing the ^13^C polarization levels in PPYV (*P*_13C_=2 %) and PG (*P*_13C_=2 %). For these phenyl‐functionalized α‐ketocarboxylates, it appears that the hydride exchange rates remain too fast compared to substrate exchange rates and polarization transfer dynamics. Future optimization attempts to reduce hydride exchange rates while maintaining substrate exchange rates (e. g. by altering the catalyst's properties) may hold promise for higher polarization levels.

Additionally, we show that temperature dependence, *T*_1_ relaxation, and polarization buildup studies are effective methods for estimating substrate and hydride exchange rates for understanding SABRE dynamics. The detailed measurements were fit with an improved kinetic model. This new model has been improved by using a spin‐dynamics derived pumping term (Eq. 6), merging kinetics, as used before,^[57]^ with spin dynamics as discussed in the SI. The fitted model represents the data well and gives clues for future optimization of hyperpolarization levels for these α‐ketocarboxylate systems.

Taken together, our results show that SABRE is a robust method that is broadly applicable to hyperpolarizing α‐ketocarboxylates in a site‐specific manner. A number of these substrates, once ^13^C labeled, are promising candidates for *in vivo* imaging.

## Conflict of Interests

The authors declare the following competing financial interest(s): E.Y.C. and B.M.G. declare a stake of ownership in XeUS Technologies LTD. T.T., E.Y.C., and P.M.T. declare a stake of ownership in Vizma Life Sciences (VLS). VLS is developing products related to the research being reported. T.T. and E.Y.C. serve on the Scientific Advisory Board (SAB) of VLS. The terms of this arrangement have been reviewed and approved by NC State University in accordance with its policy on objectivity in research.

4

## Supporting information

As a service to our authors and readers, this journal provides supporting information supplied by the authors. Such materials are peer reviewed and may be re‐organized for online delivery, but are not copy‐edited or typeset. Technical support issues arising from supporting information (other than missing files) should be addressed to the authors.

Supporting Information

## Data Availability

The data that support the findings of this study are available from the corresponding author upon reasonable request.
