# Supplementary material for: Carbon‐13 Hyperpolarization of α‐Ketocarboxylates with Parahydrogen in Reversible Exchange
Source: ChemMedChem. 2024 Dec 10;20(5):e202400378. doi: 10.1002/cmdc.202400378 (PMC11884822; doi:10.1002/cmdc.202400378)
Supplement: Supplementary file 1 — Supporting Information [file CMDC-20-e202400378-s001.pdf]

ChemMedChem

Supporting Information

Carbon-13 Hyperpolarization of α-Ketocarboxylates with Parahydrogen in Reversible Exchange

Stephen J. McBride,* Keilian MacCulloch, Patrick TomHon, Austin Browning, Samantha Meisel, Mustapha Abdulmojeed, Boyd M. Goodson, Eduard Y. Chekmenev, and Thomas Theis*

Supporting Information for

Carbon-13 Hyperpolarization of α-Ketocarboxylates with Parahydrogen in Reversible Exchange

Stephen J. McBride,*[a] Keilian MacCulloch,[a] Patrick TomHon,[a,b] Austin Browning,[a] Samantha Meisel,[a] Mustapha Abdulmojeed,[a] Boyd M. Goodson,[c] Eduard Y. Chekmenev,[d] and Thomas Theis*[a,e]

[a] *Department of Chemistry, North Carolina State University, Raleigh, North Carolina, 27695, United States*
[b] *Vizma Life Sciences, Chapel Hill, North Carolina, 27514, United States*
[c] *School of Chemical & Biomolecular Sciences and Materials Technology Center, Southern Illinois University, Carbondale, IL, 62901, United States*
[d] *Department of Chemistry, Integrative Biosciences (Ibio), Karmanos Cancer Institute (KCI), Wayne State University, Detroit, Michigan, 48202, United States*
[e] *Department of Physics, North Carolina State University, Raleigh, North Carolina, 27695, United States*

Table of Contents

1 Polarization Calculations S2
2 Oxaloacetate Degradation S4
3 Relaxation, Exchange, and Polarization Buildup Fitting S6
4 Polarization Pumping Term Derivation S9
5 Polarization Buildup Fitting Parameters S11
6 Parahydrogen-Derived Hydride and Substrate Linewidths S12

1 Polarization Calculations

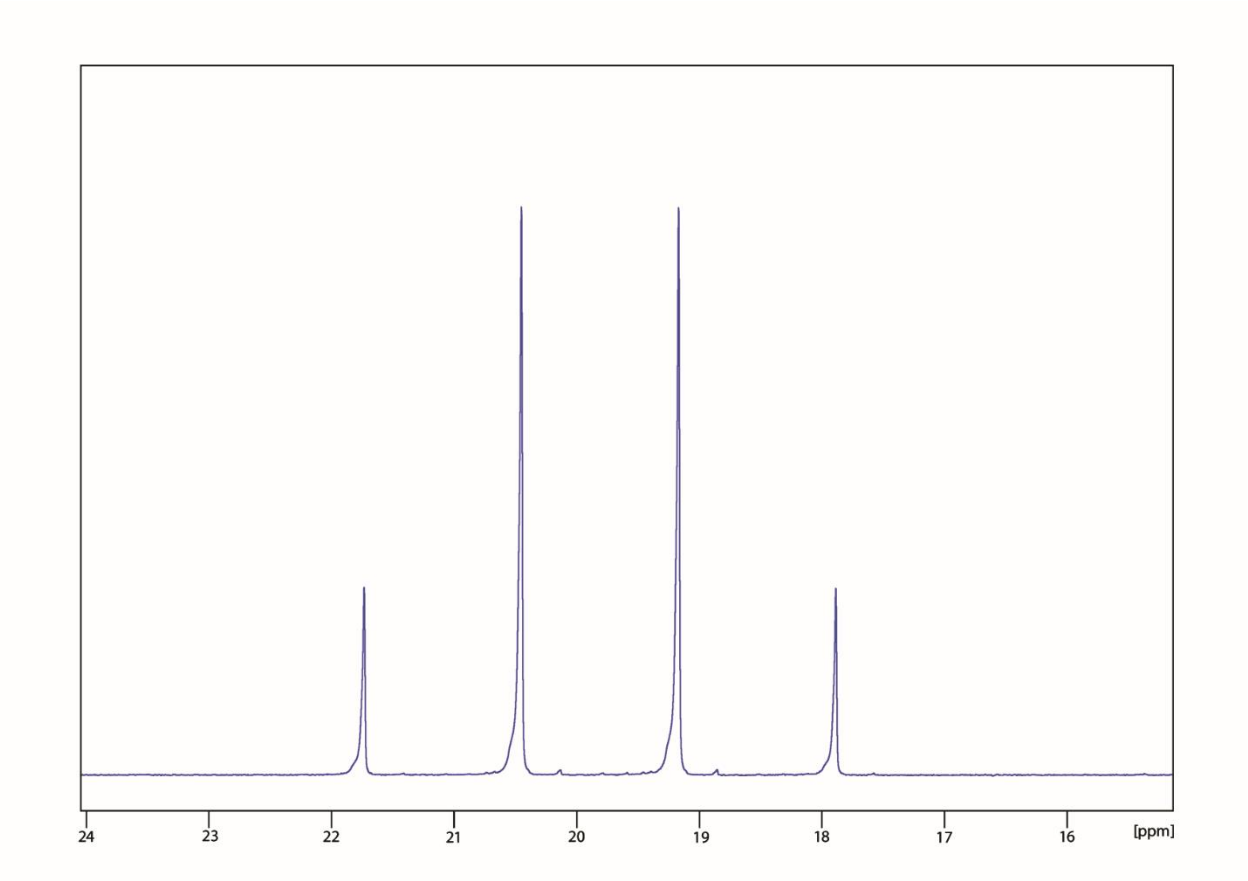

Figure S1. Single ^{13}C scan of pure [2-^{13}C]ethyl acetate thermalized at 9.4 T and 298 K for ^{13}C polarization calculations.

A reference spectrum of pure [2-^{13}C]-ethyl acetate, thermally polarized at 9.4T (**Figure S1**) was used to calculate ^{13}C Polarization using the following function:

$$P = tanh\left(\frac{\gamma B_0 \hbar}{2k_B T}\right) * \frac{S_{HP}}{S_{REF}} * \frac{C_{REF}}{C_{HP}} * \frac{SA_{REF}}{SA_{HP}} * 100\% \quad \textbf{Eq. S1}$$

The tanh term calculates the thermal ^{13}C polarization of the standard at a given magnetic field (9.4 T) and temperature (298 K). The second term contains S_{HP} and S_{REF} which refer to the integrated signals of the hyperpolarized and reference samples. The third term contains C_{REF} and C_{HP} which refer to the concentrations of the reference sample (10.2 M) and hyperpolarized samples. Hyperpolarized sample concentrations were 24 mM for substrate free in solution and 6 mM for substrate bound to the polarization transfer catalyst, which assumes that every catalyst molecule has one substrate bound to it. The fourth term

takes into account the differences in sensitive volume by comparing the surface area of the reference sample (SA_{REF}) to the surface area of the hyperpolarized sample (SA_{HP}). This term is necessary as the hyperpolarized sample contained a capillary tube within the sensitive volume of the sample, whereas the thermal reference standard contained no capillary. This term was determined to be 1.326 in a previous publication using the same parahydrogen bubbling apparatus.[1]

2 Oxaloacetate Degradation

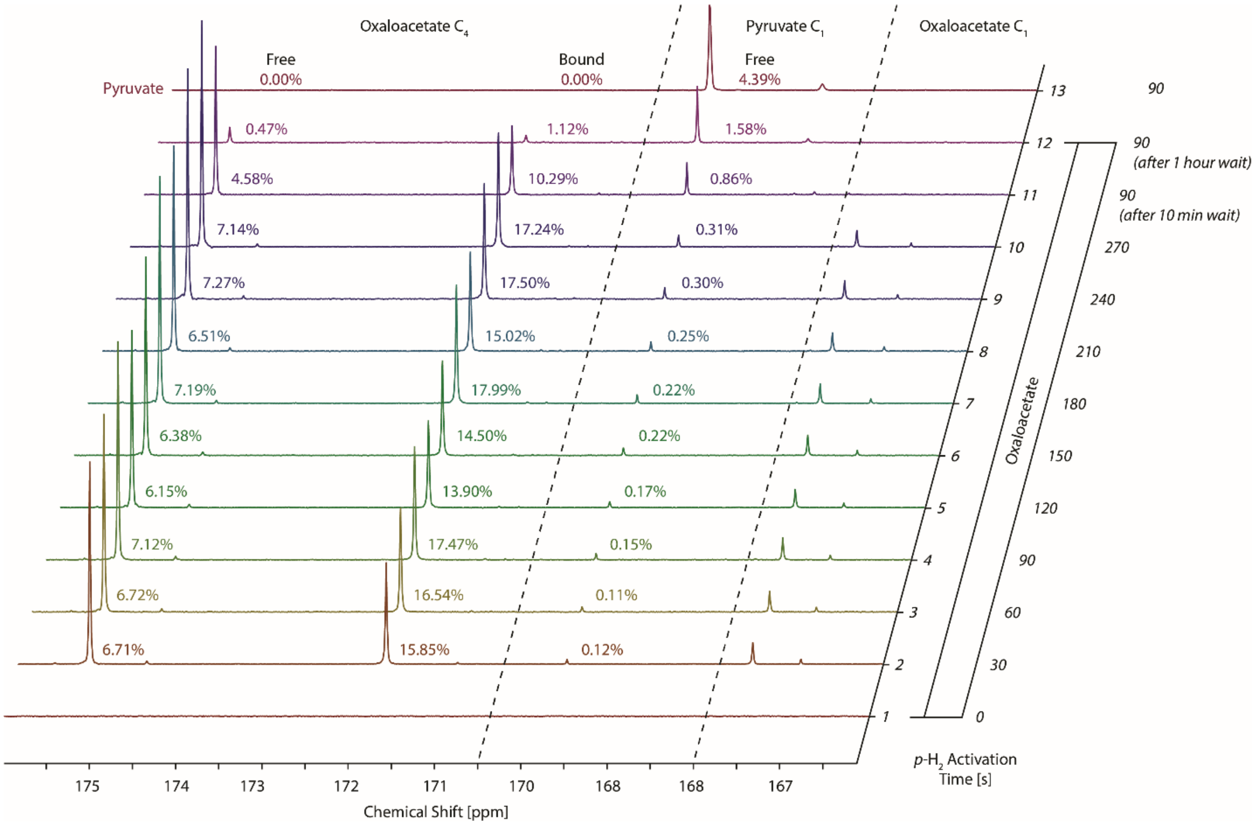

Figure S2. Oxaloacetate C_1 and C_4 and pyruvate C_1 polarization as a function of cumulative p-H_2 bubbling time at room temperature. Note that between spectra 10 and 11, the sample was unperturbed for 10 minutes, followed by 90 s of p-H_2 bubbling. Between spectra 11 and 12, the sample was unperturbed for 60 minutes, followed by 90 s of p-H_2 bubbling. Spectrum 13 reflects a separate pyruvate sample after 90 s of p-H_2 bubbling at room temperature.

While performing temperature dependence, relaxation, and polarization buildup dynamic studies for oxaloacetate, we noted a number of inconsistencies with regards to peak locations and signal intensities. Reports from a prior work indicated that oxaloacetate in its deprotonated form rapidly decarboxylated into pyruvate and carbon dioxide. Additionally, this study noted an increase in the rate of decarboxylation in the presence of metals.[2] To assess that oxaloacetate was decarboxylating in our SABRE system, we performed a series of temperature-cycling experiments with an initial temperature of 0 °C in which we increased the cumulative parahydrogen bubbling time into our solution at SABRE conditions. All data was acquired using samples containing 6 mM SABRE pre-catalyst, 30 mM α-ketocarboxylate substrate, and 24 mM DMSO in 600 μL of methanol-d_4. For oxaloacetate, a 1:1 ratio of NaOH in methanol-d_4 was added to deprotonate the

carboxylic acid group. After each addition of parahydrogen, the sample was transferred into a 9.4 T spectrometer for signal detection.

A comparison of the oxaloacetate spectra to a standard SABRE-hyperpolarized pyruvate spectrum (Spectrum 13) shows that after 30 seconds of parahydrogen addition (Spectrum 2), oxaloacetate has already begun to degrade into pyruvate (0.12% free pyruvate C_1 polarization, 6.71% free oxaloacetate C_4 polarization). After 270 seconds of cumulative parahydrogen addition (Spectrum 10), the sample has further degraded into pyruvate (0.31% free pyruvate C_1 polarization, 7.14% free oxaloacetate C_4 polarization). After allowing the sample to rest for 60 minutes without parahydrogen addition and subsequent 90 second addition of parahydrogen (Spectrum 12), the oxaloacetate has almost entirely degraded into pyruvate (1.58% free pyruvate C_1 polarization, 0.47% free oxaloacetate C_4 polarization).

3 Relaxation, Exchange, and Polarization Buildup Fitting

Low-field (0.3 μT) T_1 relaxation and polarization buildup at 0.3 μT were measured to characterize the SABRE dynamics of each α-ketocarboxylate. All α-ketocarboxylate samples were prepared and activated as indicated in the main text. For each low-field T_1 measurement, the samples were first cooled to 0 °C (-10 °C for PG) in the bore of the spectrometer. The samples were then shuttled out of the spectrometer and into a polarization transfer field (PTF) set to 0.3 μT with no active cooling where the sample temperature can equilibrate with ambient conditions. While in the PTF, p-H_2 was bubbled into the sample for 90 s (15 s for PG) at 100 psi and 75 sccm. After a variable delay with the sample remaining at 0.3 μT, the sample was shuttled back into the bore of the magnet for signal detection at high field. After detection, the sample temperature was equilibrated to 0 °C (-10 °C for PG) before the next experiment. To analyze this T_1 relaxation data, the data was first fit to a mono-exponential decay function:

$$P_x[t] = P_0 e^{\frac{-(t-t_0)}{T_1}} \qquad \textbf{Eq. S2}$$

and the initial free and bound polarization ($P_F[0]$ and $P_B[0]$) were extracted and presented below in **Table S1**.

	$P_F[0]$	$P_B[0]$
pyruvate	10.724	12.476
2-oxobutyrate	14.016	22.530
alpha-ketoglutarate	1.766	2.343
phenylgyloxylate	1.290	0.481
phenylpyruvate	0.478	0.417

Table S1. Initial free and bound polarization values extracted for each α-ketocarboxylate extracted from the low field (0.3 μT) T_1 relaxation data fit to a monoexponential decay function (**Eq. S2**)

The full T_1 relaxation data was then fit to a two-state (Free-Bound) SABRE model where P_F and P_B are the free and bound substrate ^{13}C polarization, ρ_F and ρ_B are the free and bound substrate relaxation rates, and k is the substrate exchange rate. The system of differential equations that describes this two-state SABRE system[1] was:

$$\frac{dP_F}{dt} = kP_B[t] - (k + \rho_F)P_F[t]$$
$$\frac{dP_B}{dt} = kP_F[t] - (k + \rho_B)P_B[t] \qquad \textbf{Eq. S3}$$

This system of differential equations was then given boundary conditions for initial free and bound polarization extracted from the mono-exponential fits and analytically solved. The T_1 relaxation data for both the free and bound substrate was then simultaneously fit to both functions using MultiNonLinearModelFit in Wolfram Mathematica and values for ρ_F, ρ_B, and k are extracted. These values, the raw T_1 relaxation data, and the fit curves are presented in **Figure 4** of the main text.

To model polarization buildup, a related set of differential equations was used with the addition of a polarization pumping term, Γ, to describe the spin order transfer from the parahydrogen-derived hydrides to the $^{13}C_1$ nucleus on the α-ketocarboxylate substrate.

$$\frac{dP_F}{dt} = kP_B[t] - (k + \rho_F)P_F[t]$$
$$\frac{dP_B}{dt} = \Gamma + kP_F[t] - (k + \rho_B)P_B[t] \qquad \textbf{Eq. S4}$$

Γ is derived from the level anti-crossing of a 3-spin system, here between the two parahydrogen-derived hydrides and the primarily C_1 target spin of the α-ketocarboxylate. The derivation of the pumping term, Γ, is detailed in **Section 4** of this document and is as follows,

$$\Gamma = \frac{\pi^2 \tau_{life} \Delta J_{CH}^2}{1 + \pi^2 \tau_{life}^2 \left(2\Delta J_{CH}^2 + 4\left(-J_{HH} + \frac{\Sigma J_{CH}}{4} + \Delta\nu_{CH} \right)^2 \right)} \qquad \textbf{Eq. S5}$$

where τ_{life} is the lifetime of the parahydrogen-derived hydrides, J_{HH} is the *J*-coupling between the parahydrogen-derived hydrides, $\Delta J_{CH} = (J_{CH'} - J_{CH})$, $\Sigma J_{CH} = (J_{CH'} + J_{CH})$, and $\Delta\nu_{CH} = (\nu_H - \nu_C)$. For all α-ketocarboxylate systems examined, $\Delta J_{CH} = 0.8$, $\Sigma J_{CH} = 0$, $\Delta\nu_{CH} = 11.85$, and $J_{HH} = -11.85$.

As the lifetime of the parahydrogen-derived hydrides (τ_{life}) is dependent on the exchange rate of these hydrides, the solution temperature change over the course of the experiment impacts this lifetime and exchange rate. To characterize this effect on τ_{life}, the Arrhenius equation was used, where *p*, *a*, and *g* are fitting parameters:

$$\tau_{life}[t] = \frac{1}{k_{hydride}} = \frac{1}{p + a * e^{\frac{-g}{T[t]}}} \qquad \textbf{Eq. S6}$$

A description of the τ_{life} term and the extracted fitting parameters for p, a, and g are shown in **Section 5** of this document.

To solve the set of differential equations describing polarization buildup, boundary conditions of 0 for initial free and bound polarization were applied to the analytical solution. The parameters c and d are used to scale P_B and P_F, respectively. The polarization buildup data for both the free and bound substrate was then simultaneously fit to both functions using MultiNonLinearModelFit in Wolfram Mathematica and values for p, a, and g were extracted. These values are presented in **Section 5** of this document. The raw polarization buildup data and fit curves are presented in **Figure 5** of the main text.

4 Polarization Pumping Term Derivation

Unlabeled α-ketocarboxylate SABRE systems can be represented as an AA'B system where A and A' are the parahydrogen-derived hydrides and B is the target ^{13}C spin. AA' are represented in the singlet-triplet basis and B is represented in the Zeeman basis. The complete Hamiltonian that describes this 3-spin system is an 8x8 matrix. However, of specific interest is the block of the Hamiltonian that connects the parahydrogen-derived $|S_0\rangle$ state to ^{13}C states. In this block, the $|S_0\beta\rangle \rightarrow |T_{-}\alpha\rangle$ transition describes the primary SABRE spin transfer mechanism. This 2x2 block of the Hamiltonian that connects these spin states is as follows:

$$\hat{H} = 2\pi \begin{pmatrix} -J_{HH} + \frac{\Sigma J_{CH}}{4} & -\frac{\Delta J_{CH}}{2\sqrt{2}} \\ -\frac{\Delta J_{CH}}{2\sqrt{2}} & -\Delta\nu_{CH} \end{pmatrix} \begin{matrix} |S_0\beta\rangle \\ |T_{-}\alpha\rangle \end{matrix} \quad \text{(columns: } |S_0\beta\rangle, |T_{-}\alpha\rangle\text{)}$$ **Eq. S7**

When the diagonal elements of this 2x2 matrix are equivalent, spin order transfer occurs, resulting in an overpopulation of the $|\alpha\rangle$ state on the target ^{13}C nuclei. Equivalence between the diagonal elements is achieved by setting the magnetic field such that:

$$\Delta\nu_{CH} = J_{HH} - \frac{\Sigma J_{CH}}{4}$$ **Eq. S8**

Where $\Delta J_{CH} = (J_{CH'} - J_{CH})$, $\Sigma J_{CH} = (J_{CH'} + J_{CH})$, and $\Delta\nu_{CH} = (\nu_H - \nu_C)$. When the diagonal elements of this 2x2 matrix are equivalent, the off-diagonal elements can drive population into the $|\alpha\rangle$ state on the target ^{13}C nuclei. When **Eq. S8** is solved for magnetic field where $\Delta\nu_{CH} = (\gamma_H - \gamma_C)B_0$, we obtain:

$$B_0 = \frac{J_{HH} - \frac{\Sigma J_{CH}}{4}}{(\gamma_H - \gamma_C)}$$ **Eq. S9**

For the C_1 spin in α-ketocarboxylates, this occurs at ≈0.3 µT. When parahydrogen is freshly supplied to the SABRE system, our initial state is described by the following density matrix:

$$\rho[0] = \begin{pmatrix} 1 & 0 \\ 0 & 0 \end{pmatrix}$$ **Eq. S10**

This density matrix is propagated with the Hamiltonian of **Eq. S7** according to the Liouville-Von Neumann equation:

$$\rho[t] = e^{i\hat{H}t}\rho[0]e^{-i\hat{H}t} \qquad \textbf{Eq. S11}$$

And obtains the following equation in the (2,2) matrix element, representing the population in $|\alpha\rangle$ over time without exchange:

$$P[t] = \frac{8\Delta J_{CH}^2 \sin\left(\frac{1}{4}\pi t\sqrt{8\Delta J^2 + (-4J_{HH} + 4\Delta\nu_{CH} + \Sigma J_{CH})^2}\right)^2}{8\Delta J_{CH}^2 + (-4J_{HH} + 4\Delta\nu_{CH} + \Sigma J_{CH})^2} \qquad \textbf{Eq. S12}$$

To derive our pumping term Γ[t], we also have to account for exchange dynamics of this system. Therefore, we integrate over the polarization lifetime t with and average lifetime τ_{life} and normalize the function by τ_{life}^2, to take into account two things. First, division by τ_{life} normalizes with respect to the exponential weighting function $e^{-\frac{1}{\tau_{life}}}$ function, where $\int_0^\infty e^{-\frac{1}{\tau_{life}}}dt = \tau_{life}$; and the second division by τ_{life} takes into account that the number of turnovers (i.e. the number of pumping events per unit time) is directly is directly proportional to $\frac{1}{\tau_{life}}$:

$$\Gamma[\mathrm{t}] = \frac{1}{\tau_{life}^2}\int_0^\infty P[t]e^{-\frac{1}{\tau_{life}}}dt \qquad \textbf{Eq. S13}$$

As the end result, after integration, we obtain the polarization pumping term Γ[t] as:

$$\Gamma[t] = \frac{\pi^2\tau_{life}\Delta J_{CH}^2}{1 + \pi^2\tau_{life}^2\left(2\Delta J_{CH}^2 + 4\left(-J_{HH} + \frac{\Sigma J_{CH}}{4} + \Delta\nu_{CH}\right)^2\right)} \qquad \textbf{Eq. S14}$$

We note that for the α-ketocarboxylate systems, the hydride exchange limits τ_{life} because the hydride exchange is faster than the substrate exchange. Therefore, $\frac{1}{\tau_{life}} = k_H$, the hydride exchange rate.

5 Polarization Buildup Fitting Parameters

The polarization pumping term (Γ) from **Eqs. 5-7** in the main body of text contains a term representing the lifetime of parahydrogen in the catalytically active SABRE species τ_{life}. This lifetime is a function of time as described below:

$$\tau_{life}[t] = \frac{1}{k_{hydride}} = \frac{1}{p + a * e^{\frac{-g}{T[t]}}} \quad \textbf{Eq. S15}$$

where:

$$T[t] = T_0 + \frac{\Delta T}{\Delta t} t \quad \textbf{Eq. S16}$$

For pyruvate, 2-oxobutyrate, alpha-ketoglutarate, and phyenylpyruvate, $T_0 = 273.15K$ and $\frac{\Delta T}{\Delta t} = \frac{25}{600}$. For phenylglyoxylate, $T_0 = 263.15K$ and $\frac{\Delta T}{\Delta t} = \frac{35}{600}$. After fitting the polarization buildup data as described in the manuscript, the following fitting parameters were obtained:

	PYV		2-OB		AKG	
	Free	Bound	Free	Bound	Free	Bound
p (s^{-1})	8.05×10^3	-2.53×10^5	-6.56×10^4	-4.01×10^6	-1.78×10^5	-4.50×10^3
a (s^{-1})	3.96×10^4	3.77×10^6	6.68×10^4	4.07×10^6	1.85×10^5	8.57×10^4
g (K)	978	641	4.67	4.11	10.7	802

	PG		PPYV	
	Free	Bound	Free	Bound
p (s^{-1})	-3.64×10^3	-7.64×10^3	-2.21×10^3	-5.73×10^3
a (s^{-1})	5.14×10^7	6.50×10^{10}	1.27×10^6	4.59×10^7
g (K)	2.51×10^3	4.20×10^3	1.67×10^3	2.47×10^3

Table S2. Summary of fitting terms for the polarization buildup curves extracted using the process detailed in **Section 3** of this document.

6 Parahydrogen-Derived Hydride and Substrate Linewidths

Linewidths of ^{1}H and ^{13}C Signals corresponding to the catalytically active SABRE species for each α-ketocarboxylate were obtained at a number of starting temperatures in temperature-cycling SABRE experiments as described in the main body of the text.

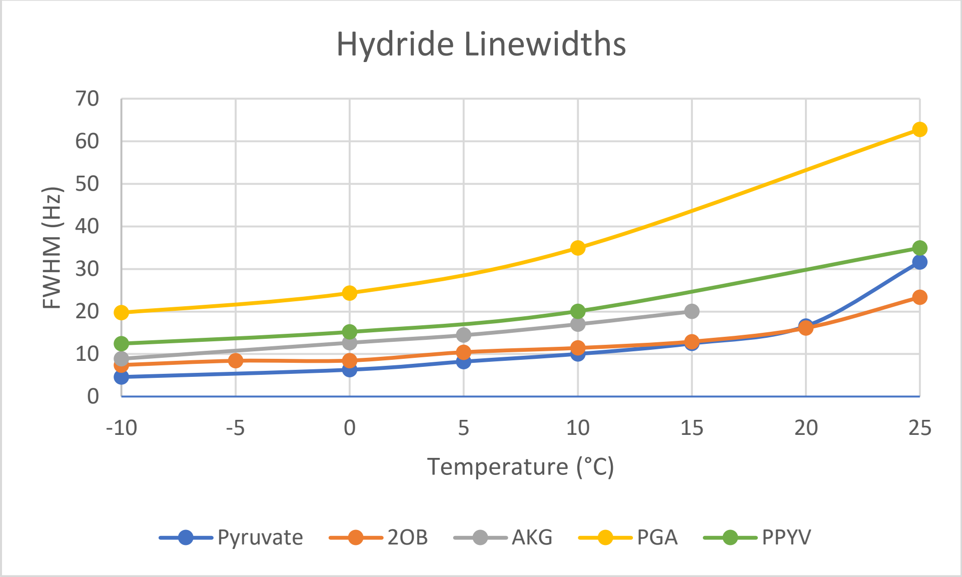

Figure S3. ^{1}H Linewidths for the parahydrogen-derived hydrides of complex **3b** at different initial temperatures.

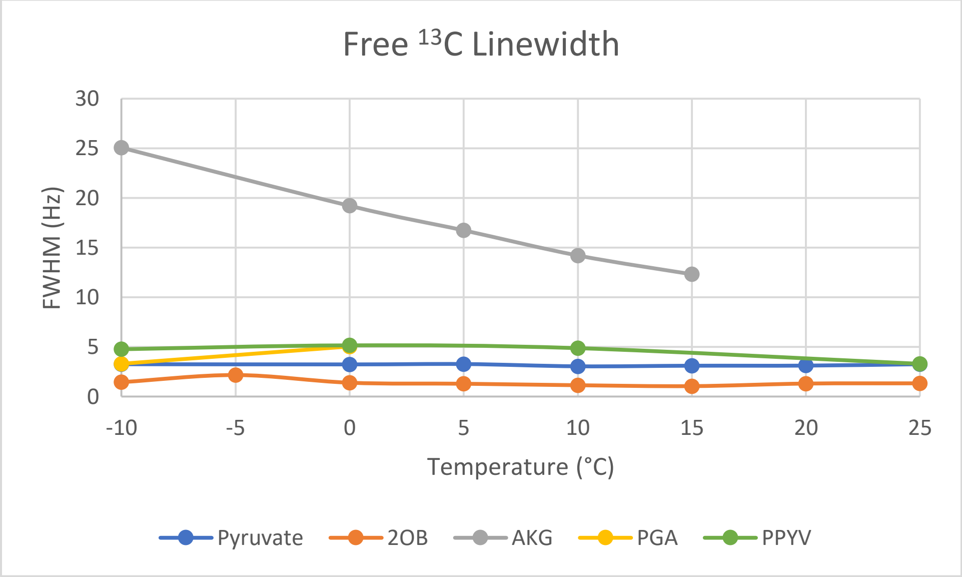

Figure S4. ^{13}C Linewidths for each α-ketocarboxylate unbound to the catalyst at different initial temperatures. Incomplete data sets arise from there being no measurable ^{13}C polarization at the respective starting temperature.

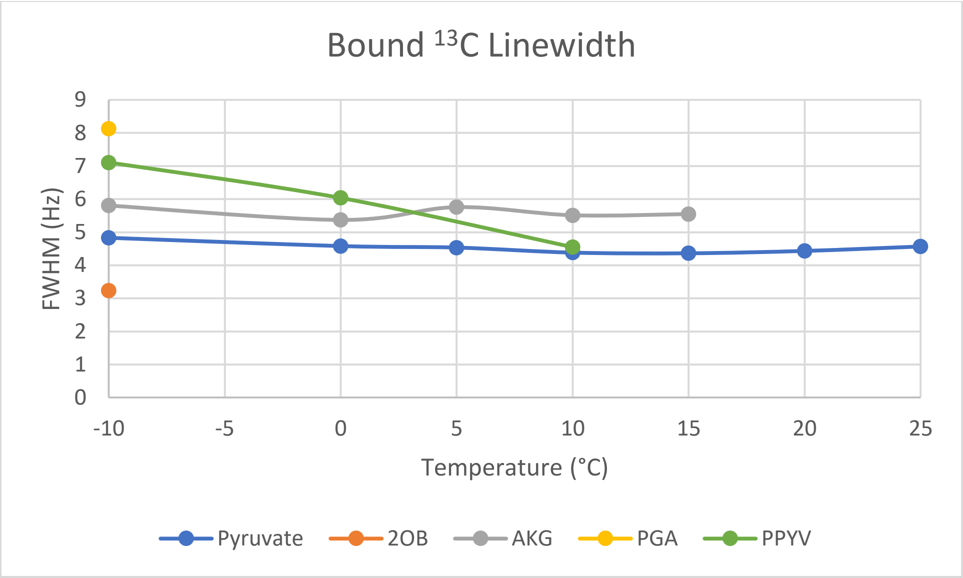

Figure S5. ^{13}C Linewidths for each α-ketocarboxylate bound to the catalyst in the **3b** complex at different initial temperatures. Incomplete data sets arise from there being no measurable ^{13}C polarization at the respective starting temperature.

References

1 P. Tomhon, M. Abdulmojeed, I. Adelabu, S. Nantogma, M. Shah, H. Kabir, S. Lehmkuhl, E. Y. Chekmenev and T. Theis, *J Am Chem Soc*, 2022, **144**, 282–287.

2 L. C. Kurz, J. J. H. Ackerman and G. R. Drysdale, *Biochemistry*, 1985, **24**, 452–457.
